# Contrasting outcomes of inherited *NOD2* loss-of-function variants on immunotherapy response in cancer

**DOI:** 10.1073/pnas.2525911122

**Published:** 2025-12-05

**Authors:** Nora Bousdar, Elisabeth Pérez-Ruiz, Isabel Barragán, José Carlos Benítez, Antonio Rueda-Domínguez, Javier Oliver

**Affiliations:** ^a^Servicio de Oncología Médica, Hospital Regional de Málaga, Grupo B-05 Instituto de Investigación Biomédica de Málaga y Plataforma en Nanomedicina, Málaga 29009, Spain; ^b^Servicio de Oncología Médica, Hospital Virgen de la Victoria, Grupo B-05 Instituto de Investigación Biomédica de Málaga y Plataforma en Nanomedicina, Málaga 290010, Spain

We have read with great interest the recent article by Barnet et al. (1), which reported in Non-Small Cell Lung Cancer (NSCLC) patients a significantly higher frequency (30%) of *NOD2* loss-of-function (LOF) variants in exceptional anti–PD-1/PD-L1 responders with immune-related adverse events (irAEs) > 2. This work expands our understanding of how inherited germline variants may help predict immunotherapy response and adverse events in lung cancer.

However, we wish to share some observations from a study conducted in our laboratory on a Mediterranean cohort, which revealed a distinct spectrum and opposite outcomes, despite using a similar approach to that of Barnet’s study. We analyzed 117 metastatic NSCLC patients treated with anti–PD-1/PD-L1 at two Spanish centers (2019–2024), assessing toxicity following Common Terminology Criteria for Adverse Events (CTCAE v5) and response at 3, 6, and 12 mo, as well as germline variation by whole exome sequencing plus imputation.

**Fig. 1.**
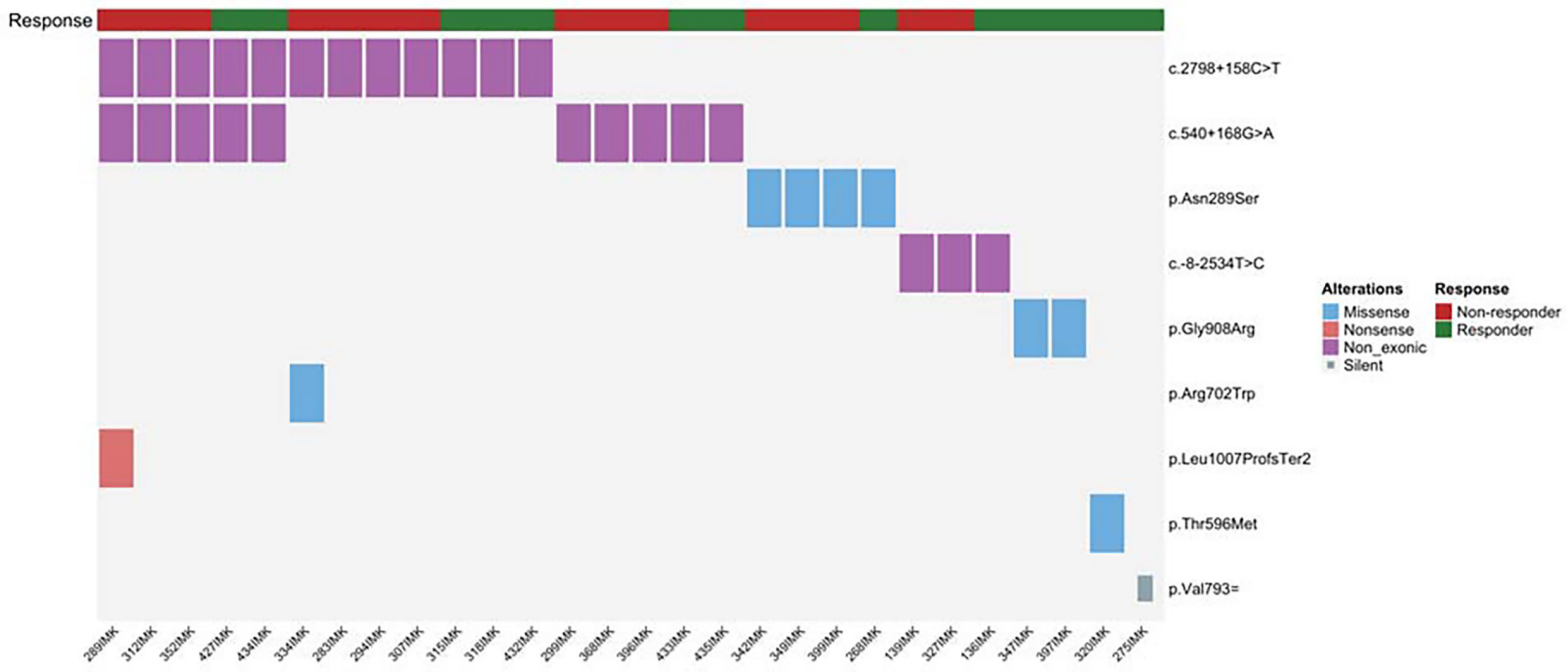
Displays the spectrum of *NOD2* genetic variants detected in our NSCLC patients and their immunotherapy response status. The analysis reveals a heterogeneous pattern of variants, including missense alterations (such as p.Asn289Ser, p.Arg702Trp), nonsense mutations (e.g., p.Gly908Arg), nonexonic changes, and silent variants. The response classification demonstrates variable outcomes, with some variants such as p.Gly908Arg present only in responder patients, while classical LOF variants p.Arg702Trp and p.Leu1007ProfsTer2 are found exclusively in nonresponder patients to immunotherapy.

Focusing on *NOD2* gene (Fig. 1), we identified *NOD2* variants in 32 patients (27.35%), a frequency nearly identical to that reported by Barnet. However, *NOD2* variant carriers in our cohort generally showed poor treatment responses: Only 34.48% of *NOD2*-variant carriers in our cohort-maintained response at 12 mo, compared with the 70 to 80% exceptional response rates reported by Barnet. Moreover, using their 24-mo Progression-Free Survival (PFS) criterion, we project that only 15 to 20% of our *NOD2*-variant carriers would qualify as exceptional responders, a fourfold to fivefold lower rate than their findings.

Moreover, LOF variants were more frequent in our nonresponders, including p.Leu1007ProfsTer2 (fs1007), contrasting with the exceptional responses observed in the Mel1 patient in Barnet´s report. In contrast, our responder group exclusively carried exonic variants, classified as benign or of uncertain significance, such as p.V908L, p.T596M, and p.V793=, which were not found by Barnet et al. We also evaluated intronic/regulatory *NOD2* variants, being rs5743289 (c.2798+158C>T) (2) the most prevalent in our cohort and a strong expression Quantitative Trait Loci (eQTL) associated with significantly reduced *NOD2* transcript levels (eQTLGen Z = −21.8) (3). However, in our data, neither LOF variants nor this eQTL showed significant association with clinical benefit or improved outcomes.

These discrepancies highlight the multifaceted nature of genetic influence, emphasizing the roles of population stratification, genetic diversity, multifactorial determinants of immunotherapy response, and the context of immune-related adverse events as critical modifiers. In line with the literature, our findings indicate that the clinical impact of *NOD2* variants may be population-specific (4). Furthermore, mechanistic evidence suggests that the influence of NOD2 on immunotherapy outcomes may be modulated by host–microbiome interactions (5). Recent data reinforce that diet-driven and geography-dependent features of the gut microbiome critically shape immunotherapy response, with distinct microbial signatures of benefit identified across different cohorts and countries (6). These results call for a more nuanced understanding of NOD2’s role, considering diverse genetic, environmental, and therapeutic landscapes.

All in all, we believe that these findings refine the role of germline variants in cancer immunotherapy and show how contrasting results from independent cohorts are essential for advancing personalized immunotherapy strategies.
